# Association between Micronutrient Intake and Breast Cancer Risk According to Body Mass Index in South Korean Adult Women: A Cohort Study

**DOI:** 10.3390/nu14132644

**Published:** 2022-06-26

**Authors:** Huiyeon Song, Ansun Jeong, Thi Xuan Mai Tran, Jiseon Lee, Mikyung Kim, Boyoung Park

**Affiliations:** 1 Graduate School of Public Health, Hanyang University, Seoul 04763, Korea; shy101704@hanyang.ac.kr (H.S.); ansun_0315@naver.com (A.J.); 2 Department of Preventive Medicine, Hanyang University College of Medicine, Seoul 04763, Korea; maitran@hanyang.ac.kr (T.X.M.T.); easypink92@gmail.com (J.L.); kmkkim@hanyang.ac.kr (M.K.); 3 Department of Medicine, Hanyang University College of Medicine, 222 Wangsimni-ro, Seoul 04763, Korea

**Keywords:** breast cancer, body mass index, micronutrients, vitamin B6, vitamin C

## Abstract

This study investigated the association between micronutrient intake and breast cancer risk in South Korean adult women. This association was stratified according to body mass index (BMI) categories. Data from the Korean Genome and Epidemiology Study (KoGES) and the Health Examinee Study were analyzed. Altogether, 63,337 individuals (aged ≥40 years) completed the baseline and first follow-up surveys; 40,432 women without a history of cancer at baseline were included in this study. The association between micronutrient intake and breast cancer was determined by estimating the hazard ratio (HR) and 95% confidence interval (CI) using the Cox proportional hazard regression model. A stratified analysis by BMI (<25 kg/m^2^ and ≥25 kg/m^2^) was performed. The an analysis of 15 micronutrients and breast cancer risk revealed that none of the micronutrients were associated with breast cancer risk after adjusting for covariates. In obese women, the risk of breast cancer was significantly reduced in the group that consumed vitamin C more than the recommended level (HR = 0.54, 95% CI: 0.31–0.93) and vitamin B6 levels above the recommended level (HR = 0.48, 95% CI: 0.25–0.89). In obese women, exceeding the recommended daily intake levels of vitamin C and vitamin B6 was associated with a lower risk of breast cancer. However, other micronutrients were not associated with breast cancer risk in these women.

## 1. Introduction

According to GLOBOCAN 2020, breast cancer is the most common cancer in women worldwide and ranks first in terms of cancer incidence and mortality in most countries (159 and 110 countries, respectively) [1]. According to the 2018 cancer registration statistics in South Korea, breast cancer is the most common cancer in women, with 23,647 (20.5%) cases, and the incidence rate has been continuously increasing [2]. Known risk factors for breast cancer are mainly reproductive factors, including the age at birth, the number of births, breastfeeding experience and duration, and the use of hormone replacement therapy after menopause [3]. The factors related to lifestyle, the level of physical activity, and alcohol consumption are well-known risk factors; however, diet findings have not been clearly understood [4,5]. In a meta-analysis, data from several studies showed that the intake of vitamins A and E reduced breast cancer risk, but no significant results were found in the cohort study [6]. The total retinol intake and breast cancer risk were not significant in the case-control study but were significant in the cohort study [6]. A study on the association between grain intake and breast cancer risk revealed heterogeneity [7]. A case-control study showed that grain intake reduced the risk of breast cancer; however, this finding was not significant in a cohort study [7].

The incidence of breast cancer in women is rapidly increasing in Asian countries, including in South Korea [8]. The rapid westernization of diet and lifestyle has been suggested to increase the incidence of breast cancer [1]. Westernized eating habits such as a high-fat diet [9,10], a high processed meat intake [11,12], a low fruit/vegetable intake [13,14], and soy products [15,16] have been associated with an increased breast cancer incidence in randomized controlled trials or meta-analyses of observational studies [17]. There is evidence of the protective effect of micronutrients on breast cancer progression through the alteration of signaling pathways related to apoptosis, the suppression of proliferation, and the invasion of breast cancer cells in in vitro models [18]. However, epidemiological studies have mostly focused on specific food items or macronutrients, and epidemiological studies on micronutrients and breast cancer are limited.

In South Korea, most studies on diet and cancer have focused on single nutrients or macronutrients, and most of the results were retrieved from small case-control studies, showing large variations in study results [19]. In addition, different associations between nutrition and breast cancer, such as the protective association of soy isoflavon in Asians, but no association in Western populations [15,16], were observed due to a large variation in the amount of intake between countries. Therefore, the association between micronutrients and breast cancer needs to be assessed in a large-scale prospective study, reflecting the dietary pattern of each country. Thus, we assessed the association between micronutrient intake and breast cancer risk in a large prospective cohort in South Korea from the Korean Genome and Epidemiology Study (KoGES) cohort study.

## 2. Materials and Methods

### 2.1. Study Design and Population

The KoGES is a cohort study administered by the Korea Agency for Disease Control and Prevention for the purpose of identifying the genetic and environmental etiology of common chronic diseases in the Korean population to establish a scientific basis for personalized prevention. The KoGES consists of six subcohorts: three population-based cohorts and three gene-environment model studies [20]. Of the six subcohorts, the data of the Health Examinee (HEXA) study, the largest subcohort, was applied in this study. The details of the KoGES and HEXA studies have been described previously [20].

**Figure 1:**
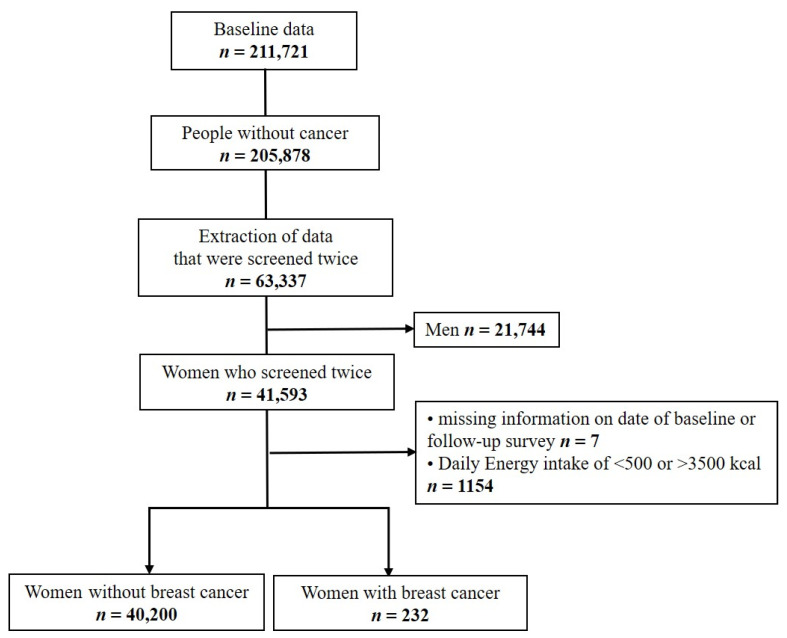
Flow chart for the participant selection protocol.

The baseline study population of the HEXA study included men and women aged ≥40 years who underwent a national health examination at 38 health examination centers from 2004 to 2013 [21]. Of the 211,721 participants who completed the baseline survey, those who reported a past history of cancer diagnosed by a doctor at the baseline survey (*n* = 5843) were excluded (Figure 1). A total of 63,337 individuals who completed the follow-up survey from 2012 to 2016 were considered for this study. Only women (*n* = 41,593) were included in the study. Women with missing information on the date of the baseline or follow-up survey and women with a daily energy intake of <500 kcal or >3500 kcal (*n* = 1161) were excluded, leaving 40,432 participants to be included in the analysis. This study was approved by the institutional review board of the Hanyang University College of Medicine, Republic of Korea (approval no. HYUIRB-202106-003-1).

### 2.2. Assessment of Nutrient Intake

Dietary evaluation in the KoGES was based on the results of a standardized semi-quantitative food frequency questionnaire (FFQ). A total of 103 items were included in the FFQ, and micronutrient intake was estimated based on the FFQ. The detailed process for the development and validation of the FFQ is described elsewhere [22]. The estimated micronutrient intake in the HEXA study was divided according to the 2020 Dietary Reference Intakes for Koreans (KDRIs) established by the Ministry of Health and Welfare in Korea [23]. The KDRIs provide the recommended daily nutritional intake for 15 micronutrients (calcium, phosphorus, iron, potassium, vitamin A, sodium, vitamin B1_,_ vitamin B2, niacin, vitamin C, zinc, vitamin B6, folate, vitamin E, and cholesterol) and four main nutrients (energy, proteins, fats, and carbohydrates) according to age group. Participants were divided into two groups: women who consumed more than the recommended daily nutritional intake and those who consumed up to the recommended daily nutritional intake. Cut-off values differ by age group (30–49 years, 50–64 years, 65–74 years, and ≥75 years) and by nutrients. For example, women aged 30–49 years consuming >700 mg of calcium fall into the recommended excess group (>recommended intake). Details on the recommended nutrient intake standards are presented in Table A1.

### 2.3. Breast Cancer Incidence

The incidence of breast cancer was defined as that among those who reported cancer-free at baseline survey and those who answered that they had been diagnosed with breast cancer by a doctor after the baseline survey at the follow-up survey. A total of 232 women reported that they had been diagnosed with breast cancer by a physician after the baseline survey.

### 2.4. Covariates

Participants’ information was retrieved from the data surveyed by well-trained staff using standardized questionnaires. Basic survey data included sociodemographic factors, medical history, family medical history, smoking, drinking, level of physical activity, and female reproductive history [20]. The covariates considered in this study were age (40–49, 50–59, 60–69, and ≥70 years), body mass index (BMI; <25 kg/m^2^, ≥25 kg/m^2^, or missing), history of benign breast tumors (no, yes, or missing), age at menarche (<15 years, ≥15 years, or missing), menopausal status and age (no, <52 years, ≥52 years, or missing), breastfeeding (no, yes, or missing), smoking (never, former, current, or missing), drinking (no, yes, or missing), physical activity (no, <150 min/week, ≥150 min/week, or missing), and family history of breast cancer (no, yes, or missing). BMI was calculated by dividing weight by the square of height in meters and was classified as ≥25 kg/m^2^ or <25 kg/m^2^.

### 2.5. Statistical Analysis

The distribution of risk factors for breast cancer and micronutrient intake at baseline was compared between women who developed breast cancer and those who did not develop. Nutritional intake was presented as the mean and proportion of women who consumed more or less than the daily recommended intake. We investigated the association between micronutrient intake (more than the recommended level vs. less than the recommended level) and breast cancer risk by estimating hazard ratio (HR) and 95% confidence interval (CI) using the Cox proportional hazards regression model adjusted for the above-mentioned covariates. The follow-up period was calculated as the number of years between the baseline survey and the year of cancer diagnosis or the year of the follow-up survey. In addition, the association between micronutrient intake and breast cancer risk was estimated in obese (BMI ≥25 kg/m^2^) and non-obese women (BMI <25 kg/m^2^). All statistical analyses were performed using the SAS statistical software (version 9.4; SAS Institute, Cary, NC, USA).

## 3. Results

Of the 40,432 cancer-free women who completed both the baseline and follow-up surveys, 232 were diagnosed with breast cancer. The mean follow-up period was 4.9 years (median: 4.0 years). Table 1 presents the participants’ characteristics. In the baseline survey, 35.0% of the women who did not develop breast cancer were in their forties, whereas 44.8% of women who developed breast cancer were in their forties, showing a younger age at baseline of women who developed breast cancer (*p* = 0.012). The proportion of premenopausal women, women without breastfeeding experience, and women with physical activity for ≥150 min/week were higher in women who developed breast cancer than those in women without breast cancer (*p* < 0.05).

**Figure 2:**
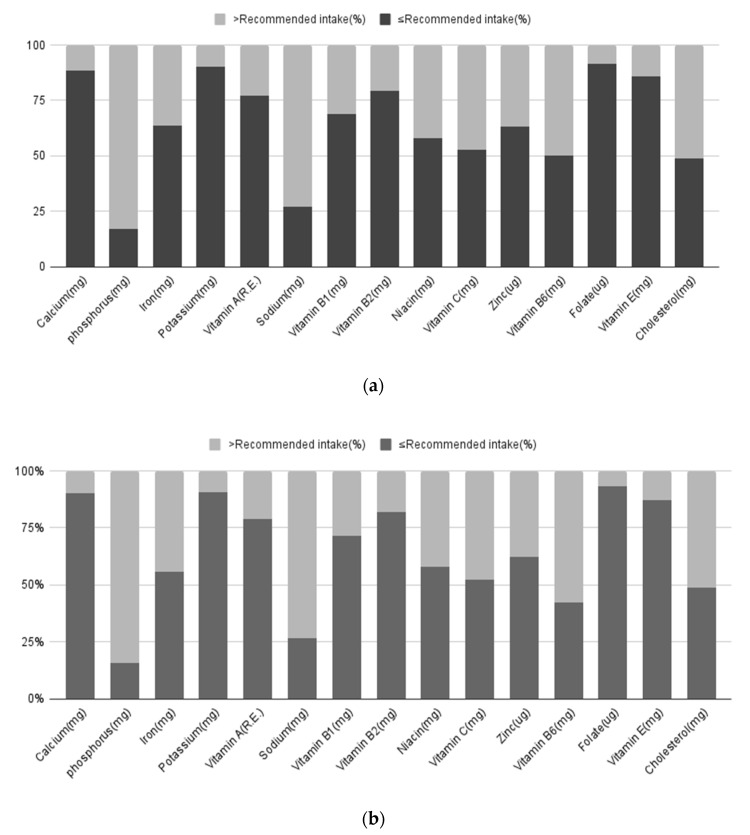
Recommended intake levels of nutrients for breast cancer (%). (**a**) Normal people; (**b**) People with breast cancer.

Table 2 compares the mean micronutrient intake and the proportion of women who consumed more or less than the daily recommended intake in those who did and did not develop breast cancer. The recommended micronutrient intakes and the distribution of breast cancer incidences are shown in Figure 2. A higher proportion of women who consumed less than the recommended amount of iron was observed in women who developed breast cancer (63.8%) than in those without breast cancer (56.0%) (*p* = 0.017). However, the average intake between the two groups was not significantly different.

Among the micronutrients, iron intake above the recommended daily intake was associated with a reduced risk of breast cancer in the univariate analysis (cHR = 0.72; 95% CI: 0.55–0.95, Table 3); however, no statistical significance was observed after adjusting for covariates. No other micronutrient showed a statistically significant association.

In a separate analysis by obesity status (BMI ≥25 kg/m^2^ or <25 kg/m^2^), iron intake more than the daily recommended level was associated with a reduced risk of breast cancer with a cHR of 0.69 (95% CI: 0.50–0.96) in a univariate analysis with a normal weight. In addition, the intake of vitamin C and vitamin B6 above the daily recommended value was associated with a reduced risk in obese women with aHR of 0.53 (95% CI: 0.30–0.92) and 0.45 (95% CI: 0.24–0.86), but a significant association was not observed in women with normal weight (Table 4). None of the other micronutrients exhibited a significant association in both women with a normal weight and obese women.

## 4. Discussion

This study evaluated micronutrients in a large prospective cohort study in Korea and identified that the intake of vitamin C and vitamin B6 above the daily recommendation level was associated with a reduced risk of breast cancer in obese women. To the best of our knowledge, this is the first prospective study to investigate the association between micronutrients and breast cancer in Korea.

Vitamin C has antioxidant activity and is found in vegetables and fruits. Its ability to reduce free radicals and oxidative damage to DNA are suggested mechanisms for reducing cancer risk [24,25]. Despite the protective association between vitamin C and several types of cancer from meta-analyses, including colorectal adenoma [26], gastric cancer [27,28], esophageal cancer [29], endometrial cancer [30], lung cancer [31], and breast cancer [6,32], several aspects should be considered.

First, the association between vitamin C and breast cancer was affected by the study design. A meta-analysis of case-control studies showed a protective effect of high-dose vitamin C on breast cancer risk; cohort studies did not show significant associations. In addition, the heterogeneity of the included studies was high [6,32]. Similarly, significant differences in the plasma levels of vitamin C between breast cancer cases and controls were observed only in case-control studies, but not in cohort-based studies [33]. Patients with breast cancer changed their dietary habits soon after the diagnosis by eating more fruits and vegetables, taking dietary supplements, and eating less meat and fat intake [34]. Therefore, the protective effect of vitamin C may be overestimated in case-control studies. A previous meta-analysis performed a subgroup analysis by menopausal status, vitamin C source, geographical location, and study design [6,32,33]. In this study, we did not find a different result according to menopausal status (Table A2), but when stratified by obesity status, a protective association was evident in obese women. Obesity is associated with higher estrogen levels in postmenopausal women due to the aromatase change of testosterone to estrogen in adipose tissue, as well as chronic inflammation status with increased oxidative stress permanently [35,36]. Thus, the antioxidant effect of vitamin C may be more prominent in women with obesity.

Second, dietary vitamin C was associated with a decreased risk of breast cancer, but supplementation did not show this association [6,32]. However, studies have shown inconsistent results regarding fruit and vegetable intake and breast cancer. A previous study showed that fruit intake was not associated with breast cancer, but a higher vegetable intake had a protective effect against breast cancer [14]. Otherwise, a meta-analysis of prospective studies showed an association between a high intake of fruit or fruit and vegetables combined with breast cancer but not with that of vegetables [13]. A recent large prospective study also showed a reduced association with an increased fruit intake [37]. Although fruits and vegetables are major sources of vitamin C, vitamin C is not prevalent in all vegetables and fruits [38]. Thus, the effect of vitamin C on breast cancer needs to be interpreted in combination with its source. In this study, vitamin C level was measured using the FFQ of 103 food items, and dietary supplements were not included in the food items. There is a moderate relationship between plasma vitamin C and estimated vitamin C from FFQ, and this relationship is stronger in non-smokers and obese people [39,40]. In this study, approximately 97% of women were non-smokers; thus, the association would reflect dietary vitamin C intake, with a better relationship with vitamin C.

Vitamin B6 is a B vitamin found in various sources, such as meat, fish, dairy, and root vegetables. Vitamin B6 is a one-carbon metabolism-related vitamin that affects carcinogenesis and development through its effect on DNA replication, repair, gene expression, DNA synthesis, and methylation, upholding DNA integrity, and regulating gene expression [41,42]. Vitamin B6 intake and serum pyridoxal 5-phosphate, an active form of vitamin B6, have been suggested to have protective effects against gastrointestinal cancers [43] including colorectal [44] and pancreatic cancers [45]. However, the association between vitamin B6 intake, serum pyridoxal 5-phosphate, and breast cancer showed inconsistent findings, even in a meta-analysis. A meta-analysis including prospective and case-control studies showed no association between dietary intake of vitamin B6 and breast cancer risk, but a protective association between serum pyridoxal 5-phosphate, an active form of vitamin B6, and breast cancer in postmenopausal women with a dose–response relationship [46]. Another meta-analysis showed a protective association between dietary intake of vitamin B6 and breast cancer but no association between pyridoxal 5-phosphate and breast cancer [43]. A recent meta-analysis of prospective studies identified a slightly reduced risk of breast cancer by 6% (pooled OR = 0.94) associated with a high intake of vitamin B6 [47]. However, in the subgroup analysis according to intake assessment, study design, menopausal status, and hormone receptor status, no significant association was observed [47]. Among the biomarkers of vitamin B6, pyridoxal 5-phosphate showed a strong correlation with vitamin B6 intake, and the correlation was affected by personal and lifestyle factors, including sex and menopausal status [48]. This might explain the inconsistencies between the studies. In this study, a protective association of high vitamin B6 intake was observed only in obese women [49]. Studies have suggested that vitamin B6 is also involved in insulin resistance by controlling the expression of adipogenesis-related genes [50]. Thus, the protective effect may be more prominent in women with obesity.

Previous studies on diet and cancer risk in Korea were mostly case-control studies [19]. To the best of our knowledge, this is the largest prospective study to show the association between micronutrients and breast cancer risk in Korean women. The main limitation of this study is that micronutrient intake was estimated based on a single assessment of the FFQ. Thus, measurement errors would be present due to the limited inclusion of food items and an inaccurate recall of their food intake over a long period [20]. Despite these limitations, the FFQ is the most common and practical dietary assessment tool used in prospective studies [20] and the FFQ applied in the KoGES has been well validated [51]. Second, cancer development was assessed using a follow-up questionnaire. Despite possible information bias, a previous study showed that self-reported cancer history in the HEXA study had a high accuracy, especially for breast cancer [52]. Third, the proportion of women who participated in the follow-up survey was low; thus, the selection bias was due to systemic differences between women who did or did not participate in the follow-up. However, when we compared the baseline characteristics between the two groups, no differences were identified. Fourth, we did not consider the subtypes of breast cancer based on hormone receptor status because of the unavailability of information. Fifth, there was only one follow-up survey, and the average follow-up period of 4.9 years may be too short to examine the effect of dietary intake on breast cancer incidence. In addition, possible confounding factors that were not measured or were measured suboptimally cannot be excluded. Therefore, it is necessary to study many patients with breast cancer with further follow-up.

In this study, only two micronutrients showed a reduced association with breast cancer risk. Recent studies have identified that only a few nutrients or food items are associated with breast cancer [37,53]. According to the report of the World Cancer Research Fund, all diet and nutrition, except for alcohol, have limited evidence on breast cancer risk [54]. These findings would support the suggestion that diet in mid-life or recent years might contribute minimally to breast cancer development [55]. However, micronutrients with a significantly reduced breast cancer risk in obese women in this study (vitamins C and B6) were intensively investigated in the Western population, which was consistent with findings in the Korean population despite variations in eating habits. Further studies related to nutritional status throughout the life course or critical period, such as during childhood or early adulthood, are warranted.

## Figures and Tables

**Table 1:** General characteristics of participants according to breast cancer.

Characteristic	Total	%	Breast Cancer				*p*-Value *
			No (*n* = 40,200)	%	Yes (*n* = 232)	%	
**Age (years)**							
40–49	14,187	35.1	14,083	35.0	104	44.8	0.012
50–59	17,471	43.2	17,392	43.3	79	34.1	
60–69	8409	20.8	8362	20.8	47	20.3	
≥70	365	0.9	363	0.9	2	0.8	
**Body mass index (kg/m^2^)**							
<25	29,103	72.0	28,939	72.0	164	70.7	0.855
≥25	11,310	27.9	11,242	27.9	68	29.3	
Missing	19	0.1	19	0.1	0	0	
**Diagnosis of benign breast cancer**							
No	28,924	71.5	28,772	71.6	152	65.5	0.061
Yes	2183	5.4	2164	5.4	19	8.2	
Missing	9325	23.1	9264	23.0	61	26.3	
**Age at menarche (years)**							
<15	15,082	37.3	14,981	37.3	101	43.6	0.140
≥15	24,468	60.5	24,341	60.6	127	54.7	
Missing	882	2.2	878	2.1	4	1.7	
**Menopausal status and age (years)**							
No	14,831	36.7	14,736	36.7	95	41.0	<0.001
<52	15,287	37.8	15,224	37.9	63	27.2	
≥52	8182	20.2	8130	20.2	52	22.4	
Missing	2132	5.2	2110	5.2	22	9.4	
**Breastfeeding**							
No	4980	12.3	4935	12.3	45	19.4	<0.001
Yes	33,497	82.9	33,326	82.9	171	73.7	
Missing	1955	4.8	1939	4.8	16	6.9	
**Smoking**							
No	39,078	96.7	38,856	96.7	222	95.7	0.106
Former	461	1.1	455	1.1	6	2.5	
Current	704	1.7	702	1.8	2	0.9	
Missing	189	0.5	187	0.4	2	0.9	
**Drinking**							
No	27,635	68.4	27,473	68.3	162	69.8	0.888
Yes	12,607	31.1	12,538	31.2	69	29.8	
Missing	190	0.5	189	0.5	1	0.4	
**Physical activity (min/week)**							
No	18,723	46.3	18,634	46.4	89	38.4	0.002
<150	4932	12.2	4910	12.2	22	9.5	
≥150	15,458	38.2	15,352	38.2	106	45.7	
Missing	1319	3.3	1304	3.2	15	6.4	
**Family history of breast cancer**							
No	6734	16.7	6701	16.7	33	14.2	0.375
Yes	553	1.4	548	1.4	5	2.2	
Missing	33,145	81.9	32,951	81.9	194	83.6	

* *p*-values for categorical variables were calculated using the chi-square test.

**Table 2:** Comparison of nutrient intake recommendations for patients with breast cancer.

Nutrient	Breast Cancer				*p*-Value *
	No (*n* = 40,200)		Yes (*n* = 232)		
	N	%	N	%	
**Calcium (mg)**					
Mean ± SD	453.46 ± 250.64		481.60 ± 311.53		0.171
≤Recommended intake **	36,307	90.3	205	88.4	0.316
>Recommended intake **	3893	9.7	27	11.6	
**Phosphorus (mg)**					
Mean ± SD	870.53 ± 319.01		886.96 ± 360.40		0.489
≤Recommended intake **	6450	16.0	40	17.2	0.621
>Recommended intake **	33,750	84.0	192	82.8	
**Iron (mg)**					
Mean ± SD	9.83 ± 4.49		10.11 ± 5.03		0.402
≤Recommended intake **	22,514	56.0	148	63.8	0.017
>Recommended intake **	17,686	44.0	84	36.2	
**Potassium(mg)**					
Mean ± SD	2229.23 ± 990.89		2277.75 ± 1077.79		0.457
≤Recommended intake **	36,382	90.5	209	90.1	0.829
>Recommended intake **	3818	9.5	23	9.9	
**Vitamin A (R.E.)**					
Mean ± SD	468.86 ± 321.25		490.82 ± 361.00		0.356
≤Recommended intake **	31,664	78.8	179	77.2	0.550
>Recommended intake **	8536	21.2	53	22.8	
**Sodium (mg)**					
Mean ± SD	2370.63 ± 1319.25		2391.46 ± 1387.47		0.811
≤Recommended intake **	10,764	26.8	63	27.2	0.897
>Recommended intake **	29,436	73.2	169	72.8	
**Vitamin B1 (mg)**					
Mean ± SD	0.96 ± 0.37		0.96 ± 0.39		0.795
≤Recommended intake **	28,657	71.3	160	69.0	0.436
>Recommended intake **	11,543	28.7	72	31.0	
**Vitamin B2 (mg)**					
Mean ± SD	0.89 ± 0.41		0.92 ± 0.45		0.305
≤Recommended intake **	32,964	82.0	184	79.3	0.288
>Recommended intake **	7236	18.0	48	20.7	
**Niacin (mg)**					
Mean ± SD	13.84 ± 5.28		13.98 ± 5.66		0.692
≤Recommended intake **	23,292	57.9	135	58.1	0.939
>Recommended intake **	16,908	42.1	97	41.9	
**Vitamin C (mg)**					
Mean ± SD	110.51 ± 68.07		114.28 ± 76.84		0.456
≤Recommended intake **	21,115	52.5	122	52.6	0.985
>Recommended intake **	19,085	47.5	110	47.4	
**Zinc (µg)**					
Mean ± SD	7.65 ± 3.05		7.78 ± 3.20		0.488
≤Recommended intake **	25,007	62.2	147	63.4	0.717
>Recommended intake **	15,193	37.8	85	36.6	
**Vitamin B6 (mg)**					
Mean ± SD	1.55 ± 0.62		1.56 ± 0.64		0.746
≤Recommended intake **	18,816	46.8	116	50.0	0.331
>Recommended intake **	21,384	53.2	116	50.0	
**Folate (µg)**					
Mean ± SD	216.96 ± 114.93		220.17 ± 116.86		0.672
≤Recommended intake **	37,554	93.4	212	91.4	0.212
>Recommended intake **	2646	6.6	20	8.6	
**Vitamin E (mg)**					
Mean ± SD	7.94 ± 3.92		8.34 ± 4.14		0.121
≤Recommended intake **	35,078	87.3	199	85.8	0.500
>Recommended intake **	5122	12.7	33	14.2	
**Cholesterol (mg)**					
Mean ± SD	161.10 ± 110.61		166.87 ± 134.66		0.515
≤Recommended intake **	19,583	48.7	113	48.7	0.998
>Recommended intake **	20,617	51.3	119	51.3	

* *p*-values for continuous and categorical variables were calculated using the *t*-test and chi-square test, respectively. ** The recommended nutrient intake standards were presented in Table A1.

**Table 3:** HR (95% CI) of breast cancer risk for recommended nutrient intake.

Nutrient	Breast Cancer		
	No. of Events/ Person Year	cHR (95% CI)	aHR * (95% CI)
**Calcium (mg)**			
≤Recommended intake **	205/177,782	1	1
>Recommended intake **	27/20,390	1.17 (0.78–1.75)	1.12 (0.72–1.76)
**Phosphorus (mg)**			
≤Recommended intake **	40/31,774	1	1
>Recommended intake **	192/166,398	0.92 (0.65–1.29)	0.88 (0.59–1.32)
**Iron (mg)**			
≤Recommended intake **	148/111,063	1	1
>Recommended intake **	84/87,109	0.72 (0.55–0.95)	0.74 (0.52–1.06)
**Potassium (mg)**			
≤Recommended intake **	209/177,987	1	1
>Recommended intake **	23/20,185	0.99 (0.65–1.53)	0.96 (0.59–1.57)
**Vitamin A (R.E.)**			
≤Recommended intake **	179/155,182	1	1
>Recommended intake **	53/42,990	1.08 (0.79–1.47)	1.12 (0.80–1.56)
**Sodium (mg)**			
≤Recommended intake **	63/52,688	1	1
>Recommended intake **	169/145,484	0.98 (0.73–1.30)	0.98 (0.72–1.34)
**Vitamin B1 (mg)**			
≤Recommended intake **	160/140,297	1	1
>Recommended intake **	72/57,875	1.10 (0.83–1.45)	1.14 (0.78–1.66)
**Vitamin B2 (mg)**			
≤Recommended intake **	184/160,930	1	1
>Recommended intake **	48/37,242	1.15 (0.83–1.58)	1.17 (0.80–1.73)
**Niacin (mg)**			
≤Recommended intake **	135/114,543	1	1
>Recommended intake **	97/83,629	0.99 (0.76–1.28)	0.95 (0.67–1.33)
**Vitamin C (mg)**			
≤Recommended intake **	122/102,230		
>Recommended intake **	110/95,942	0.97 (0.75–1.26)	0.95 (0.71–1.26)
**Zinc (µg)**			
≤Recommended intake **	147/122,609	1	1
>Recommended intake **	85/75,563	0.94 (0.72–1.23)	0.89 (0.61–1.28)
**Vitamin B6 (mg)**			
≤Recommended intake **	116/91,707	1	1
>Recommended intake **	116/106,465	0.87 (0.67–1.12)	0.78 (0.56–1.09)
**Folate (µg)**			
≤Recommended intake **	212/184,209	1	1
>Recommended intake **	20/13,963	1.27 (0.81–2.01)	1.32 (0.80–2.19)
**Vitamin E (mg)**			
≤Recommended intake **	199/171,659	1	1
>Recommended intake **	33/26,513	1.09 (0.76–1.58)	1.07 (0.69–1.66)
**Cholesterol (mg)**			
≤Recommended intake **	113/99,875	1	1
>Recommended intake **	119/98,297	1.03 (0.79–1.33)	1.11 (0.83–1.49)

* Adjusted for energy, age (40–49, 50–59, 60–69, and ≥70 years), body mass index (<25 kg/m^2^ or ≥25 kg/m^2^), diagnosis of benign breast cancer (no or yes), age at menarche (<15 or ≥15 years), menopausal status and age (no, <52 years, or ≥52 years), breastfeeding (no or yes), smoking (never, former, or current), drinking (no or yes), physical activity (no, <150 min/week, or ≥150 min/week), and family history of breast cancer (no or yes); aHR, adjusted hazard ratio; CI, confidence interval. ** The recommended nutrient intake standards were presented in Table A1.

**Table 4:** Adjusted HR (95% CI) of breast cancer risk for recommended nutrient intake according to BMI.

Nutrient	BMI < 25 kg/m^2^		BMI ≥ 25 kg/m^2^	
	cHR (95% CI)	aHR * (95% CI)	cHR (95% CI)	aHR * (95% CI)
>Recommended Intake (Reference: ≤Recommended Intake) **				
Calcium (mg)	1.00 (0.61–1.66)	0.93 (0.53–1.62)	1.64 (0.84–3.22)	1.60 (0.74–3.47)
Phosphorus (mg)	0.83 (0.56–1.23)	0.74 (0.46–1.18)	1.21 (0.60–2.45)	1.34 (0.60–2.97)
Iron (mg)	0.69 (0.50–0.96)	0.68 (0.44–1.04)	0.78 (0.48–1.27)	0.90 (0.48–1.70)
Potassium (mg)	0.98 (0.58–1.64)	0.94 (0.53–1.69)	1.03 (0.47–2.26)	0.93 (0.38–2.31)
Vitamin A (R.E.)	1.05 (0.73–1.52)	1.09 (0.72–1.63)	1.13 (0.66–1.96)	1.17 (0.63–2.14)
Sodium (mg)	1.01 (0.71–1.42)	1.01 (0.70–1.47)	0.90 (0.52–1.54)	0.89 (0.50–1.58)
Vitamin B1 (mg)	1.15 (0.83–1.59)	1.23 (0.79–1.92)	0.99 (0.59–1.67)	0.97 (0.48–1.96)
Vitamin B2 (mg)	1.15 (0.79–1.67)	1.18 (0.74–1.88)	1.14 (0.63–2.05)	1.13 (0.55–2.35)
Niacin (mg)	1.04 (0.76–1.42)	1.02 (0.68–1.54)	0.87 (0.53–1.41)	0.80 (0.42–1.52)
Vitamin C (mg)	1.18 (0.87–1.61)	1.19 (0.85–1.67)	0.60 (0.36–0.99)	0.53 (0.30–0.92)
Zinc (µg)	0.97 (0.71–1.34)	0.93 (0.60–1.43)	0.88 (0.53–1.44)	0.79 (0.40–1.56)
Vitamin B6 (mg)	1.01 (0.74–1.37)	0.98 (0.66–1.45)	0.61 (0.37–0.98)	0.45 (0.24–0.86)
Folate (µg)	1.25 (0.73–2.17)	1.29 (0.71–2.36)	1.31 (0.57–3.03)	1.25 (0.49–3.19)
Vitamin E (mg)	1.11 (0.72–1.72)	1.08 (0.64–1.82)	1.05 (0.52–2.11)	0.98 (0.42–2.30)
Cholesterol (mg)	0.96 (0.71–1.31)	1.04 (0.73–1.47)	1.19 (0.73–1.92)	1.31 (0.76–2.26)

* Adjusted for energy, age (40–49, 50–59, 60–69, or ≥70 years), diagnosis of benign breast cancer (no or yes), age at menarche (<15 or ≥15 years), menopausal status and age (no, <52 years, or ≥52 years), breastfeeding (no or yes), smoking (never, former, or current), drinking (no or yes), physical activity (no, <150 min/week, or ≥150 min/week), and family history of breast cancer (no or yes); aHR, adjusted hazard ratio; CI, confidence interval. ** The recommended nutrient intake standards were presented in Table A1.

## Data Availability

The data underlying the results of out study are not publicly available because of KoGES data policy. Data are available from the Division of Genetic Epidemiology and Health Index, Korea Center for Disease Control and Prevention, for researchers who meet the criteria for access to confidential data.

## Appendix A

**Table A1:** Dietary reference intake levels for females in South Korean in 2020 according to age group.

Nutrient	Age Group (y)			
	30–49	50–64	65–74	≥75
Calcium (mg)	700	800	800	800
Phosphorus (mg)	580	580	580	580
Iron (mg)	14	8	8	7
Potassium (mg)	3500	3500	3500	3500
Vitamin A (R.E.)	650	600	600	550
Sodium (mg)	1500	1500	1500	1500
Vitamin B1 (mg)	1.1	1.1	1	0.8
Vitamin B2 (mg)	1.2	1.2	1.1	1
Niacin (mg)	14	14	13	12
Vitamin C (mg)	100	100	100	100
Zinc (µg)	8	8	7	7
Vitamin B6 (mg)	1.4	1.4	1.4	1.4
Folate (µg)	400	400	400	400
Fiber (g)	20	20	20	20
Vitamin E (mg)	12	12	12	12
Cholesterol (mg)	180	120	50	20

**Table A2:** Adjusted HR (95% CI) of breast cancer risk for the recommended nutrient intake level according to menopausal age.

Nutrient	No	<52	≥52
	aHR * (95% CI)	aHR * (95% CI)	aHR * (95% CI)
>Recommended Intake (Reference: ≤Recommended Intake) **			
Calcium (mg)	1.08 (0.54–2.16)	0.61 (0.22–1.65)	2.12 (0.85–5.29)
Phosphorus (mg)	1.28 (0.64–2.55)	0.98 (0.44–2.22)	0.61 (0.28–1.33)
Iron (mg)	0.73 (0.37–1.44)	0.57 (0.30–1.10)	0.87 (0.44–1.72)
Potassium (mg)	0.62 (0.25–1.51)	0.55 (0.20–1.53)	2.17 (0.88–5.36)
Vitamin A (R.E.)	1.42 (0.84–2.41)	0.60 (0.29–1.22)	1.21 (0.61–2.42)
Sodium (mg)	0.77 (0.48–1.24)	1.34 (0.70–2.57)	0.89 (0.47–1.68)
Vitamin B1 (mg)	1.02 (0.57–1.81)	0.87 (0.42–1.80)	2.31 (1.05–5.08)
Vitamin B2 (mg)	1.05 (0.56–1.95)	0.85 (0.39–1.83)	2.60 (1.21–5.59)
Niacin (mg)	1.12 (0.66–1.91)	0.78 (0.40–1.51)	1.14 (0.55–2.38)
Vitamin C (mg)	0.91 (0.58–1.42)	1.35 (0.78–2.33)	0.83 (0.45–1.54)
Zinc (µg)	0.88 (0.50–1.57)	0.89 (0.45–1.77)	0.83 (0.38–1.81)
Vitamin B6 (mg)	1.12 (0.67–1.89)	0.60 (0.31–1.13)	0.84 (0.42–1.70)
Folate (µg)	1.13 (0.46–2.75)	1.19 (0.47–3.03)	1.22 (0.39–3.79)
Vitamin E (mg)	1.27 (0.65–2.46)	0.76 (0.31–1.86)	1.06 (0.39–2.87)
Cholesterol (mg)	1.17 (0.74–1.86)	1.06 (0.60–1.87)	1.23 (0.67–2.26)

* Adjusted for energy, age (40–49, 50–59, 60–69, and ≥70 years), body mass index (<25 kg/m^2^ or ≥25 kg/m^2^), diagnosis of benign breast cancer (no or yes), age at menarche (<15 or ≥15 years), breastfeeding (no or yes), smoking (never, former, or current), drinking (no and yes), physical activity (no, <150 min/week or ≥150 min/week), and family history of breast cancer (no or yes); aHR, adjusted hazard ratio; CI, confidence interval. ** The recommended nutrient intake standards were presented in Table A1.

**Table A3:** Summary of previous studies on the association between nutrients and breast cancer risk by obesity status.

(**a**). Calcium—1/2pt		
Study type	Title	Findings
Cohort study	Calcium intake is not related to breast cancer risk among Singapore Chinese women [56].	- No association between calcium and breast cancer risk. - No findings by obesity status.
Case-control study	Dairy Products, Calcium Intake, and Breast Cancer Risk: A Case-Control Study in China [57].	- Calcium intake was associated with reduced breast cancer risk. - No findings by obesity status.
Meta-analysis	Meta-analysis of vitamin D, calcium, and the prevention of breast cancer [58].	- Calcium intake was associated with reduced breast cancer risk. - No findings by obesity status.
Case-control study	Raw and Cooked Vegetables, Fruits, Selected Micronutrients, and Breast Cancer Risk: A Case–Control Study in Germany [59].	- No association between calcium and breast cancer risk. - No findings by obesity status.
(**b**). Phosphorus		
Study type	Title	Findings
Cohort study	Imbalanced Nutrient Intake in Cancer Survivors from the Examination from the Nationwide Health Examination Center-Based Cohort [60].	- Phosphorus intake was associated with reduced breast cancer risk. - No findings by obesity status.
Case-control study	The Intake of some Nutrients is Associated with the Risk of Breast Cancer: Results from Jordanian Case-Control Study [61].	- Phosphorus intake was associated with increased breast cancer risk. - No findings by obesity status.
(**c**). Iron		
Study type	Title	Findings
Cohort study	Dietary Iron and Heme Iron Intake and Risk of Breast Cancer: A Prospective Cohort Study [62].	- No association between iron and breast cancer risk. - No findings by obesity status.
Case-control study	A case-control study on heme/non-heme iron and breast cancer risk breast cancer risk [63].	- No association between iron and breast cancer risk. - No findings by obesity status.
Case-control study	Raw and Cooked Vegetables, Fruits, Selected Micronutrients, and Breast Cancer Risk: A Case–Control Study in Germany [59].	- No association between iron and breast cancer risk. - No findings by obesity status.
Meta-analysis	Iron intake, body iron status, and risk of breast cancer: a systematic review and meta-analysis [64].	- Iron intake was associated with increased breast cancer risk. - No findings by obesity status.
(**d**). Vitamin A		
Study type	Title	Findings
Cohort study	A Prospective Study of the Intake of Vitamins C, E, and A and the Risk of Breast Cancer [65].	- Vitamin A intake was associated with reduced breast cancer risk. - No findings by obesity status.
Case-control study	Intake of Carrots, Spinach, and Supplements Containing Vitamin A in Relation to Risk of Breast Cancer [66].	- Vitamin A intake was associated with reduced breast cancer risk. - No findings by obesity status.
Meta-analysis	Vitamin A and Breast Cancer Survival: A Systematic Review and Meta-analysis [67].	- Vitamin A intake was associated with reduced breast cancer risk. - No findings by obesity status.
(**e**). Vitamin B1		
Study type	Title	Findings
Cohort study	B-Vitamin Intake from Diet and Supplements and Breast Cancer Risk in Middle-Aged Women: Results from the Prospective NutriNet-Santé Cohort [68].	- Vitamin B1 intake was associated with reduced breast cancer risk. - No findings by obesity status.
Case-control study	Nutrient Patterns and Risk of Breast Cancer among Iranian Women: a Case- Control Study [69].	- Vitamin B1 intake was associated with reduced breast cancer risk. - No findings by obesity status.
Cohort study	B-Vitamin Intake, One-Carbon Metabolism, and Survival in a Population-Based Study of Women with Breast Cancer [68].	- Vitamin B1 intake was associated with reduced breast cancer risk. - No findings by obesity status.
(**f**). Vitamin B2		
Study type	Title	Findings
Cohort study	Dietary B-Vitamin Intake and Risk of Breast, Endometrial, Ovarian and Colorectal Cancer among Canadians [70].	- Vitamin B2 intake was associated with reduced breast cancer risk. - No findings by obesity status.
Case-control study	Nutrient Patterns and Risk of Breast Cancer among Iranian Women: a Case–Control Study [69].	- Vitamin B2 intake was associated with reduced breast cancer risk. - No findings by obesity status.
Meta-analysis	Dietary vitamin B2 intake and breast cancer risk: a systematic review and meta-analysis [71].	- Vitamin B2 intake was associated with reduced breast cancer risk. - No findings by obesity status.
(**g**). Vitamin C		
Study type	Title	Findings
Cohort study	Vitamin C intake from diary recordings and risk of breast cancer in the UK Dietary Cohort Consortium [72].	- Vitamin C intake was associated with reduced breast cancer risk. - No findings by obesity status.
Case-control study	Raw and Cooked Vegetables, Fruits, Selected Micronutrients, and Breast Cancer Risk: A Case–Control Study in Germany [59].	- Vitamin C intake was associated with reduced breast cancer risk. - No findings by obesity status.
Cohort study	Dietary fiber, vitamins A, C, and E, and risk of breast cancer: a cohort study [73].	- Vitamin C intake was associated with reduced breast cancer risk. - No findings by obesity status
(**h**). Zinc		
Study type	Title	Findings
Prospective nested case-control study	Serum zinc and dietary intake of zinc in relation to risk of different breast cancer subgroups and serum levels as a marker of intake: a prospective nested case-control study [74].	- No association between zinc and breast cancer risk. - No findings by obesity status.
Case-control study	Raw and Cooked Vegetables, Fruits, Selected Micronutrients, and Breast Cancer Risk: A Case–Control Study in Germany [59].	- Zinc intake was associated with reduced breast cancer risk. - No findings by obesity status.
Meta-analysis	A meta-analysis of zinc levels in breast cancer [75].	- Zinc intake was associated with reduced breast cancer risk. - No findings by obesity status.
(**i**). Vitamin B6		
Study type	Title	Findings
Nested case-control study	Plasma Folate, Vitamin B_6_, Vitamin B_12_, Homocysteine, and Risk of Breast Cancer [76].	- Vitamin B6 intake was associated with reduced breast cancer risk. - No findings by obesity status.
Nested case-control study	Association of vitamin B6, vitamin B12 and methionine with risk of breast cancer: a dose–response meta-analysis [46].	- Vitamin B6 intake was associated with reduced breast cancer risk. - No findings by obesity status.
Meta-analysis	Folate, Vitamin B_6_, and Vitamin B_12_ Intake and the Risk of Breast Cancer Among Mexican Women [77].	- No association between vitamin B6 and breast cancer risk. - No findings by obesity status.
(**j**). Folate		
Study type	Title	Findings
Case-control study	Raw and Cooked Vegetables, Fruits, Selected Micronutrients, and Breast Cancer Risk: A Case–Control Study in Germany [59].	- Folate intake was associated with reduced breast cancer risk. - No findings by obesity status.
Meta-analysis	Higher dietary folate intake reduces the breast cancer risk: a systematic review and meta-analysis [78].	- No association between folate and breast cancer risk. - No findings by obesity status.
Meta-analysis	Folate, Vitamin B_6_, and Vitamin B_12_ Intake and the Risk of Breast Cancer Among Mexican Women [77].	- Folate intake was associated with reduced breast cancer risk. - No findings by obesity status.
(**k**). Vitamin E		
Study type	Title	Findings
Meta-analysis	Retinol, vitamins A, C, and E and breast cancer risk: a meta-analysis and meta-regression [6].	- Vitamin E intake was associated with reduced breast cancer risk. - No findings by obesity status.
Case-control study	Raw and Cooked Vegetables, Fruits, Selected Micronutrients, and Breast Cancer Risk: A Case–Control Study in Germany [59].	- No association between vitamin E and breast cancer risk. - No findings by obesity status.
(**l**). Cholesterol		
Study type	Title	Findings
Cohort study	Dietary Factors and Female Breast Cancer Risk: A Prospective Cohort Study [79].	- Cholesterol intake was associated with increased breast cancer risk. - No findings by obesity status.
Case-control study	Dietary cholesterol intake and cancer [80].	- Cholesterol intake was associated with increased breast cancer risk. - No findings by obesity status.
Meta-analysis	Updating the role of obesity and cholesterol in breast cancer [81].	- Cholesterol intake was associated with increased breast cancer risk. - No findings by obesity status.
